# Family history of later-onset breast cancer, breast healthy behavior and invasive breast cancer among postmenopausal women: a cohort study

**DOI:** 10.1186/bcr2727

**Published:** 2010-10-12

**Authors:** Robert Gramling, Timothy L Lash, Kenneth J Rothman, Howard J Cabral, Rebecca Silliman, Mary Roberts, Marcia L Stefanick, Rosanne Harrigan, Monica L Bertoia, Charles B Eaton

**Affiliations:** 1 Departments of Family Medicine and Community & Preventive Medicine, University of Rochester, 1381 South Avenue, Rochester, NY 14620, USA; 2 Department of Clinical Epidemiology, Aarhus University Hospital, Olof Palmes Alle 43-45, 8200 Aarhus N, Denmark; 3 RTI Health Solutions, RTI International, 200 Park Offices Drive, Research Triangle Park, NC 27709, USA; 4 Department of Epidemiology, Boston University School of Public Health, 715 Albany Street, Boston, MA 02118, USA; 5 Department of Biostatistics, Boston University School of Public Health, 801 Massachusetts Avenue, 3rd floor, Boston, MA 02118, USA; 6 Section of Geriatrics, Department of Medicine, Boston University School of Medicine Department of Epidemiology, Boston University School of Public Health, 88 East Newton Street, Robinson 2, Boston, MA 02118, USA; 7 Center for Primary Care & Prevention, Memorial Hospital of Rhode Island, 111 Brewster Street, Pawtucket, RI 02860, USA; 8 Department of Medicine, Stanford Prevention Research Center, Stanford University School of Medicine, Medical School Office Building, 251 Campus Drive, Mail Code 5411, Stanford, CA 94305-5411, USA; 9 Complementary and Alternative Medicine, John A. Burns School of Medicine, 651 Ilalo Street, Honolulu, HI 86821, USA; 10 Department of Community Health, Warren Alpert Medical School of Brown University, 121 S. Main Street, Box G-S121, Providence, RI 02912, USA

## Abstract

**Introduction:**

A family history of later-onset breast cancer (FHLBC) may suggest multi-factorial inheritance of breast cancer risk, including unhealthy lifestyle behaviors that may be shared within families. We assessed whether adherence to lifestyle behaviors recommended for breast cancer prevention--including maintaining a healthful body weight, being physically active and limiting alcohol intake--modifies breast cancer risk attributed to FHLBC in postmenopausal women.

**Methods:**

Breast cancer outcomes through August 2003 were analyzed in relationship to lifestyle and risk factors collected by questionnaire during enrollment (between 1993 and 1998) of 85,644 postmenopausal women into the Women's Health Initiative Observational Study.

**Results:**

During a mean follow-up of 5.4 years, 1997 women were diagnosed with invasive breast cancer. The rate of invasive breast cancer among women with an FHLBC who participated in all three behaviors was 5.94 per 1,000 woman-years, compared with 6.97 per 1,000 woman-years among women who participated in none of the behaviors. The rate among women with no FHLBC who participated in all three behavioral conditions was 3.51 per 1,000 woman-years compared to 4.67 per 1,000 woman-years for those who participated in none. We did not observe a clinically important departure from additive effects (Interaction Contrast: 0.00014; 95% CI: -0.00359, 0.00388).

**Conclusions:**

Participating in breast healthy behaviors was beneficial to postmenopausal women and the degree of this benefit was the same for women with and without an FHLBC.

## Introduction

Nearly 15% of postmenopausal women in the US report breast cancer in a first-degree relative [1]. Few women report pedigrees that are suggestive of highly penetrant, single-gene disorders such as hereditary breast and ovarian cancer [2]. Most family histories, particularly those that arise among older relatives, reflect complex risk factors representing the interaction between genes, environments, and behaviors that are often shared within families [3-5]. Under such multi-factorial inheritance conditions, it is plausible that family history is a mutable risk factor. For example, if a woman's family history arose in part because of a predominance of risk-conferring behaviors among women in her family and she adopts breast cancer-preventive behaviors, she will have ameliorated some of her risk attributed to family history.

Physical inactivity [6,7], excessive alcohol consumption [8], and patterns of energy consumption and expenditure [6,9] are modifiable behaviors that can increase the risk of breast cancer. Later-onset family history remains a risk factor for breast cancer after potential confounding by body mass index (BMI), physical activity, and alcohol consumption [5,10] is addressed. However, components of one's lifestyle are not independent of one another [11]. Earlier work has not adequately addressed the clustering of relevant behavioral states or their potential for modifying familial risk.

Women with a first-degree family member who developed breast cancer at the age of 45 years or more are referred to as having a family history of later-onset breast cancer (FHLBC). If the impact of having an FHLBC were modified by preventive behaviors, such findings would have important implications for preventive counseling of these women. This study investigates the degree to which participating in behaviors endorsed by leaders [12,13] in breast cancer prevention (physical activity, alcohol moderation, and body weight management) modifies women's breast cancer risk attributed to having an FHLBC.

## Materials and methods

### Design

We performed analyses of the Women's Health Initiative Observational Study (WHI-OS), which began in 1993. Exposure and covariate information was obtained via baseline questionnaires, blood analyses, and anthropomorphic measurements. Each participant was contacted annually to identify hospitalizations or diagnoses of diseases pertinent to the WHI-OS, including breast cancer diagnoses. All diagnoses were confirmed via medical records, diagnostic procedures, and pathology reports.

### Participants

Postmenopausal women were recruited to the WHI-OS from populations of women living near the 40 WHI clinical centers across the US. The most common method of recruitment was mass mailing to targeted groups. Women were eligible for participation in the WHI-OS if they met all of the following conditions: they were between the ages of 50 and 79 years at enrollment (1993 to 1998), they were postmenopausal, they had a life expectancy of greater than 3 years, they had no conditions that made longitudinal participation unlikely (for example, dementia, alcoholism, or major mental illness), and they were not already participating in another randomized clinical trial. For this study, additional exclusion criteria included the following: personal history of breast cancer, history of unilateral or bilateral mastectomy, or a family history of early-onset breast cancer (diagnosed before the age of 45 years) in a first-degree relative at enrollment. We excluded women in the last of these groups to maintain focus on family histories that reflect shared behaviors, environments, and genetics--and thus that plausibly are influenced by lifestyle behaviors--rather than family histories that are likely to represent genetic influences that are more dominant.

### Exposure definition

Women who reported that a mother or full sister developed breast cancer at the age of 45 years or more were defined as exposed to an FHLBC. All other women were defined as unexposed.

### Outcome definition

Cases are defined as those women who developed invasive breast cancer between enrollment (1993 to 1998) and the end of follow-up (August 2003). Case status was determined via pathology reports by a centralized WHI team of trained adjudicators. One case of invasive breast cancer was first identified at the time of death. The reported cause of death for this case was coronary heart disease. Therefore, we considered this case of breast cancer to be an incidental finding. Since autopsy was not routinely performed on all WHI-OS participants who died, we chose to censor this participant at the time of her death and not consider the event to be a case.

### Potential confounding or effect-modifying variables

Each of the following study variables was obtained by self-report on study questionnaires at baseline: age, household income, educational attainment, menstrual history, reproductive history, personal medical history, exogenous estrogen use, number of sisters, personal history of breast biopsies, usual exercise regimen, usual intake of alcoholic beverages, and smoking history. Alcohol intake was categorized as "breast healthy" if participants drank fewer than seven drinks per week. Participants were asked to report usual frequency, duration, and intensity of exercise. Exercise was categorized as "breast healthy" if participants exercised for at least 20 minutes at moderate/vigorous intensity at least five times per week. Height (without shoes) was measured at baseline. Weight (in light clothing but without shoes) was also measured. Women whose BMI was normal (between 18.5 and 24.9) at baseline measurement and who reported having maintained a BMI of less than 25 during their non-pregnant adult lifetime were categorized as maintaining a "breast healthy" body weight.

### Analyses

We described the frequency and distribution of all study variables. We categorized participants into one of four categories on the basis of their participation in the three breast healthy behaviors: exercised moderately/vigorously for at least 20 minutes at least five times per week, maintained a normal body weight, and drank no more than one alcoholic beverage per day. The range of classification was 0 (complete non-participation) to 3 (complete participation). We chose to focus on thee types of activities (exercise, weight management, and alcohol use) because these are fully endorsed as modifiable lifestyle risk factors for breast cancer by the American Cancer Society [13]. Other behavioral factors, such as fruit and vegetable intake, are described as 'uncertain risk factors'. Our classification of "breast healthy" levels of these behaviors differs from the American Cancer Society guidelines due to the way these variables were collected in the original WHI-OS data. We categorized women into dichotomous groups on the basis of the presence or absence of an FHLBC.

We performed the following primary analyses for both adherence and FHLBC to evaluate whether these factors were associated with the incidence of invasive breast cancer in the WHI-OS. We plotted cumulative hazard curves to visualize the relation, and we fit a Cox proportional hazards model to determine the hazard ratio (HR) and 95% confidence interval (CI). We confirmed proportional hazards by observing similar and non-intersecting hazard curves for each set of exposure groups.

For each of the eight strata of adherence by FHLBC, we calculated the rate of invasive breast cancer. Within the four strata of adherence, we calculated the risk attributable to FHLBC as the difference in rate between those with FHLBC and those without FHLBC, divided by the rate in those with FHLBC. To assess whether the relation between FHLBC and invasive breast cancer is modified by adherence, we compared the strata exhibiting the greatest expected contrast: complete participation and complete non-participation. For this contrast, we calculated the following interaction contrast (IC):

[RBHB=1, FHLBC=1−RBHB=1, FHLBC=0]−[RBHB=0, FHLBC=1−RBHB=0, FHLBC=0]=1C;

where FHLBC = first-degree family history of later-onset breast cancer (1 = present, 0 = absent) and BHB = breast healthy behavior (1 = complete participation, 0 = complete non-participation).

The IC indicates whether the two exposures are synergistic (IC >0), antagonistic (IC <0), or independent of one another on the additive scale (IC = 0). Our judgments about the presence or absence of interaction were based on the degree to which IC differs from 0 and the precision of that estimate. As with our previous work [14], we chose to investigate interaction as a departure from additivity of effects rather than as a departure from multiplicative effects, because that assessment has a direct bearing on public-health impact. For example, this choice allows direct estimates regarding the absolute number of women (in the population) whom we would expect to be affected by the interacting effects (that is, caseload) [15].

Confounding of the relation between the outcome and either component cause (FHLBC and breast healthy behavior) can bias the crude IC [15,16]. Neither FHLBC nor breast healthy behavior was randomly assigned in this study. Therefore, we performed the following procedure for assessing confounding of the observed IC. First, we identified whether major risk factors for breast cancer were substantial confounders of the relation between either of the component causes and the incidence of invasive breast cancer. In regard to those meeting these criteria, we calculated a pooled IC across strata of the potential confounder to assess the degree to which the pooled IC differed from the crude IC.

A first-degree family history is not, in itself, the active exposure. It is merely a marker for genomic predisposition to breast cancer. Genomic risk is often identified by the occurrence of breast cancer in a sister. It is possible that a participant who has no sister surviving into adulthood has the same genomic predisposition as a woman with a known FHLBC, but this participant has no opportunity to identify this exposure through an affected sister. Therefore, we calculated an IC that was restricted to participants reporting at least one biologic sister who reached adulthood. To establish a participant's eligibility for classification of an FHLBC, we would ideally determine whether her sisters reached the age of 45 years. However, the WHI-OS dataset asks only whether a participant's sister or sisters reached adulthood.

The Memorial Hospital Committee for Human Subjects in Research approved this analysis. As required by the WHI protocol, informed consent was obtained from all study subjects.

## Results

Ninety-three thousand six hundred seventy-six women were enrolled in the WHI-OS. Of these, 5,298 women who reported being told by a physician before enrollment that they had breast cancer, 242 women who had a unilateral or bilateral mastectomy before enrollment, and 2,756 women reporting an early-onset family history of breast cancer (in a mother or sister younger than 45 years old) were excluded. (These three criteria are not mutually exclusive.) One point nine percent of participants were lost to follow-up, 2.2% stopped follow-up, and 6.1% were deceased by the end of follow-up.

Table 1 describes the characteristics of the 85,644 women meeting all eligibility criteria. Among this group, 1,997 cases of invasive breast cancer during a mean follow-up period of 5.4 years (range = 0 to 8.4 years, standard deviation = 1.4 years) were observed. Approximately 25% of the sample were over the age of 70 years at baseline, approximately 83% were of white racial/ethnic background, and approximately 39% had completed a four-year college degree. Approximately 12% reported a family history of breast cancer (in a first-degree relative at the age of 45 years or more). Eighty-seven percent of participants drank less than seven alcoholic beverages per week, 24% exercised for at least 20 minutes at moderate/vigorous intensity at least five times per week, and 23% maintained a healthy BMI. Seven percent of women participated in all three breast healthy behaviors, and 6% participated in none of the three.

**Table 1:** Postmenopausal women (50-79 years old) who were enrolled in the Women's Health Initiative Observational Study (1993-1998) and met eligibility criteria

	Family history of later-onset breast cancer?	
**Variable**	**No** **(n = 75,665)**	**Yes** **(n = 9,979)**

Age		
50-59 years	24,892	2,757
60-69 years	33,054	4,587
70-79 years	17,719	2,635
Ethnicity		
Hispanic/Latina	3,142	245
Black/African-American	6,274	663
White	62,566	8,666
American Indian/Alaskan Native	349	38
Asian/Pacific Islander	2,279	230
Other	858	105
Highest educational attainment		
No high school diploma	3,990	452
High school diploma	12,176	1,653
Some college/technical school	27,447	3,534
College degree	31,414	4,263
Household income		
Less than $35,000	27,624	3,543
$35,000 to less than $75,000	28,151	3,809
$75,000 or more	14,270	1,874
Participation in breast healthy behavior		
Complete participation (3/3)	5,506	761
Partial participation (1/3 or 2/3)	65,542	8,558
Complete non-participation (0/3)	4,617	660

Both FHLBC (HR = 1.50, 95% CI = 1.33, 1.68) and absence of breast healthy behavior (HR_complete non-participation versus complete participation _= 1.32, 95% CI = 1.03, 1.67) were associated with a greater incidence of invasive breast cancer. The rate attributable to FHLBC did not follow a dose-dependent pattern across strata of breast healthy behavior participation (Table 2), nor did we observe a clinically important degree of interaction on the additive scale in the rates of invasive breast cancer (IC = 0.00014, 95% CI = -0.00359, 0.00388; Table 2).

**Table 2:** Rate of invasive breast cancer among postmenopausal women (50-79 years old) enrolled in the Women's Health Initiative Observational Study (1993-2003)

Number of breast healthy behaviors participated in	FHLBC	Cases	Woman-years	Rate (per 10,000 woman-years)	Rate difference	Attributable proportion
All three	Yes	25	4,206	59	24	41%
	No	108	30,807	35		
Two	Yes	95	17,136	55	14	25%
	No	544	130,438	42		
One	Yes	182	29,020	63	23	36%
	No	901	225,716	40		
None	Yes	25	3,585	70	23	33%
	No	117	25,002	47		

The women are stratified by family history of breast cancer (in a first-degree relative at the age of 45 years or more) and the degree of participation in behaviors recommended for breast cancer-preventive behaviors. To focus on the comparison of greatest contrast, women exhibiting partial adherence were excluded from the following calculation: interaction contrast = [R_BHB = all three, FHLBC = 1 _- R_BHB = all three, FHLBC = 0_] - [R_BHB = none, FHLBC = 1 _- R_BHB = none, FHLBC = 0_] = 0.0001; 95% confidence interval = -0.0036, 0.0039. FHLBC, family history of later-onset breast cancer.

Age, education, white race, income, mammography adherence before enrollment, hormone therapy use at enrollment, and age at menarche were associated with adherence to the ACS guidelines. However, only the personal history of breast biopsy demonstrated a modest confounding effect on the relation between FHLBC and the incidence of breast cancer (Table 3). The pooled IC across strata of biopsy history (IC_pooled _= 0.00012, 95% CI = -0.00369, 0.00394) did not differ from the crude IC.

**Table 3:** Hazard of invasive breast cancer according to each component cause, adjusting for potential confounders, in postmenopausal women (50-79 years old) enrolled in the Women's Health Initiative Observational Study (1993-2003)

	Component one	Component two
	
	FHLBC	Breast healthy behavior
	Crude HR (95% CI)	Crude HR (95% CI)
	
	1.50 (1.33, 1.68)	0.76 (0.60, 0.97)
Potential confounders	Adjusted HR (95% CI)	Adjusted HR (95% CI)
Age	1.48 (1.31, 1.66)	0.77 (0.61, 0.99)
Education	N/A	0.75 (0.59, 0.95)
Caucasian	1.47 (1.30, 1.65)	0.77 (0.61, 0.97)
Income of less than $35,000/year	N/A	0.77 (0.60, 0.98)
No mammogram in past 2 years	1.48 (1.31, 1.67)	0.74 (0.58, 0.94)
Breast biopsy ever	1.44 (1.28, 1.63)	N/A
Current hormone therapy use	1.52 (1.35, 1.71)	0.74 (0.58, 0.94)
Menarche at less than 12 years of age	N/A	0.77 (0.61, 0.98)
Never pregnant	N/A	N/A

CI, confidence interval; FHLBC, family history of later-onset breast cancer; HR, hazard ratio; N/A, not applicable, because these factors were not associated with the component cause and thus are not potential confounding variables for the interaction contrast.

Age, white race, mammography adherence before enrollment, history of breast biopsies before enrollment, and hormone therapy use at enrollment were associated with FHLBC, but none of these factors demonstrated a confounding effect on the relation between breast healthy behavior participation and the incidence of breast cancer (Table 3).

Fifty-four thousand four hundred seventeen women reported having at least one sister reaching adulthood. When the data were restricted to these participants, the IC differed slightly from the IC among the full sample yet still remained clinically unimportant (IC = -0.00043, 95% CI = -0.00492, 0.00406).

## Discussion

We observed that adherence to recommended breast healthy behaviors (physical activity, alcohol moderation, and body weight maintenance) did not modify the breast cancer risk attributed to a family history of late-onset breast cancer among postmenopausal women. Therefore, women who participate in breast healthy behaviors appear to derive essentially the same benefits regardless of a family history of late-onset breast cancer. Neither confounding nor exposure misclassification apparently explains our null findings. To our knowledge, no earlier research has evaluated whether adherence to a cluster of behaviors modifies the relation between family history and breast cancer.

Some earlier research has addressed the interaction between family history and individual behaviors in their association with breast cancer risk [6], although interaction is uniformly assessed on the multiplicative scale in these cohort studies. However, three such studies present enough data from which we can make some comparisons with our study, which examines additive interaction.

Sellers and colleagues [17] conducted a follow-up study among 37,105 women (age range = 55 to 69 years) who were enrolled in the Iowa Women's Health Study Cohort. The authors assessed the interaction between family history and BMI. Their data yield a negligible IC of -0.0006 with a 95% CI of (-0.0017, 0.0030). Tehard and colleagues [18] evaluated the interaction between family history and physical activity among 90,509 French women (age range = 40 and 65 years) who participated in the French E3N Study cohort. The findings of these authors, as well, demonstrate a negligible departure from additive effects (IC = -0.0009, 95% CI = -0.0022, 0.0004).

Using a case-control study design among postmenopausal women from California, Carpenter and colleagues [6] evaluated the interaction between BMI and family history on the occurrence of breast cancer. Although the authors assessed interaction only on the multiplicative scale, they provide enough data to estimate interaction on the additive scale by calculating the relative excess risk due to interaction (RERI) [19]. BMI was categorized as not more than 27 kg/m^2 ^(normal) versus greater than 27 kg/m^2 ^(overweight), and family history was categorized on the basis of the presence or absence of breast cancer in either a mother or a sister. Compared with the RR estimates of the doubly unexposed group (that is, no family history and normal BMI), those of the doubly exposed group, the family history exposed group, and the overweight group are 2.52, 1.54, and 1.27, respectively. This yields an RERI of 0.71, which is greater than the negligible RERI obtained from the cohort of Sellers and colleagues (RERI = -0.15) and the estimate from our study (RERI = 0.02). However, direct comparison with our findings is limited because our exposure classifications (cluster of breast healthy behaviors and FHLBC) differ substantively from theirs.

Our study has important limitations. Although preventive behaviors are dynamic, this analysis is based on assessment at one time point. It is possible that longitudinal assessment of breast healthy behavior would identify more clearly the distinction between women engaging in sustained participation versus sustained non-participation and, thus, present a stronger contrast. However, lifestyle behaviors are habitual and become relatively stable in adulthood as compared with childhood [20]. Our measure of body weight management takes into account the women's entire adulthood, not just the time at enrollment. We believe, therefore, that it is unlikely that changes in behavior during the relatively short period of follow-up would lead to important differences in the interaction with FHLBC on breast cancer risk.

Similarly, family history is measured only at baseline in the WHI. It is likely that some of our unexposed respondents became aware of a family history during the follow-up period and therefore were misclassified as unexposed. We also expect, however, that the opportunity to influence breast cancer detection is low given the small group of women likely to be misclassified, the short interval during which diagnostic bias might occur, and the thorough diagnostic follow-up on all trial participants. Furthermore, this bias, if present, should occur in both the breast healthy and non-breast healthy strata, thus contributing the same absolute effects on the rate difference estimates and leaving the IC unaffected. We expect, therefore, that any bias due to misclassification of family history is very unlikely to have a substantial impact on our observed IC and null conclusion.

Lastly, FHLBC is only a proxy measure for a complex set of breast cancer-relevant exposures shared among families. The exact distribution of shared behaviors, environments, and genes leading to the presence of an FHLBC is likely to differ substantially from one participant to another in this study. Since habitual health behaviors are often shared within families [21-23], it is likely that mothers and sisters of women engaging breast healthy patterns of behavior were more likely to exhibit similar types of behavior as compared with mothers and sisters of women who did not engage breast healthy behavior. This pattern would lead to the possibility that breast cancers arising among relatives of breast healthy participants occurred *despite *healthy behavior, thus suggesting greater contribution of genetic and environmental factors among this group. One way to address this concern would be to assess whether a woman's risk attributable to having an FHLBC is influenced by a discordance between her behavior and that of affected relatives. However, the WHI-OS data do not include information about the behavioral norms for women's families or affected relatives and so we could not perform such analyses.

## Conclusions

Given the growing societal awareness of, and distress about, the risk of heritable breast cancer [24,25], understanding the actions that women can take to ameliorate this risk is both timely and essential. This study suggests to both public health and office-based clinicians that adherence to breast healthy behaviors (regular exercise, weight management and alcohol moderation) benefits women with or without an FHLBC but does not function to reduce FHLBC-attributable risk. Our findings, however, do not address the degree to which behaviors that are discordant from one's affected relatives might lead to risk reduction.

## Abbreviations

BHB: breast healthy behavior; BMI: body mass index; CI: confidence interval; FHLBC: family history of later-onset breast cancer; HR: hazard ratio; IC: interaction contrast; RERI: relative excess risk due to interaction; WHI: Women's Health Initiative; WHI-OS: Women's Health Initiative Observational Study.

## Competing interests

The authors declare that they have no competing interests.

## Authors' contributions

RG, TLL, KJR, HJC, RS, and CBE took part in all analyses and drafts of this manuscript. MR and MLB participated in some analyses and contributed substantially to final drafts and resubmission of this work. MLS and RH took part in data collection as part of the original WHI and contributed substantially to final drafts of this work. All authors read and approved the final manuscript.
